# Nationwide Survey of ^60^Co Teletherapy Dosimetry*

**DOI:** 10.6028/jres.080A.066

**Published:** 1976-08-01

**Authors:** Marqarete Ehrlich, Garv L. Welter

**Affiliations:** Institute for Basic Standards, National Bureau of Standards, Washington, D.C. 20234

**Keywords:** Absorbed dose, cobalt-60 gamma radiation, computation check, dose interpretation, mailings, results, therapy departments, thermoluminescence dosimeters, uncertainty, water phantom

## Abstract

The National Bureau of Standards is performing a study of the ability of radiation-therapy departments to deliver prescribed absorbed doses of ^60^Co gamma radiation to a water phantom. Batches of thermoluminescence dosimeters are mailed to participating therapy departments for irradiation under prescribed conditions. Upon return of the dosimeters, the participants’ computations are checked and the absorbed dose is evaluated from dosimeter response. The rugged dosimetry system was assembled mainly from commercial components adapted to the present requirements of relatively high flexibility of readout parameters and data-handling techniques, and of relatively high accuracy. The uncertainty in the dose interpretation inherent in the system is estimated to be about 4 percent.

In order to illustrate the type of information that can be obtained from such a study, results of the first four mailings involving tests on 114 ^60^Co gamma-ray beams are discussed. They show about 75 percent of the dose interpretations to be within 5 percent of the prescribed absorbed dose, and about 20 percent to be within 5 to 10 percent of this dose. Four dose interpretations showed discrepancies larger than 20 percent. Differences in the computations larger than 1 percent were observed in over one-half of the cases.

## 1. Introduction

As part of the endeavor to determine the impact of the calibration activities of the National Bureau of Standards (NBS) on radiation measurements throughout the United States and to pinpoint areas in need of improvement, a study is being performed of the ability of radiation-therapy departments to deliver a prescribed absorbed dose of ^60^Co gamma rays to a water phantom. ^60^Co gamma-ray teletherapy has been widespread throughout the United States for some time, with around 1000 beams presently in active use, most of them in conjunction with radiation-measurement equipment whose calibration is traceable to NBS. Therefore, ^60^Co teletherapy beams appeared to be a good target for starting a radiation measurement-assurance study of this nature. The study is open to all interested users and is to run for a total of about two-and-one-half years. By the end of this period, it is planned to have one single test completed on about two-thirds of all ^60^Co teletherapy beams in the United States.

NBS mails sets of thermoluminescence (TL) dosimeters to participating radiotherapy departments for irradiation under prescribed conditions. Upon return of the dosimeters, NBS interprets their response in terms of absorbed dose to water and informs the participants of the test results. It is the purpose of this paper to review the main features of the procedures involved in the study and of the dosimetry methods. Also, in order to illustrate the type of information that can be obtained from a study of this nature, a discussion is given of the results for the first four mailings.

## 2. Administrative Procedure

**Figure 1:**
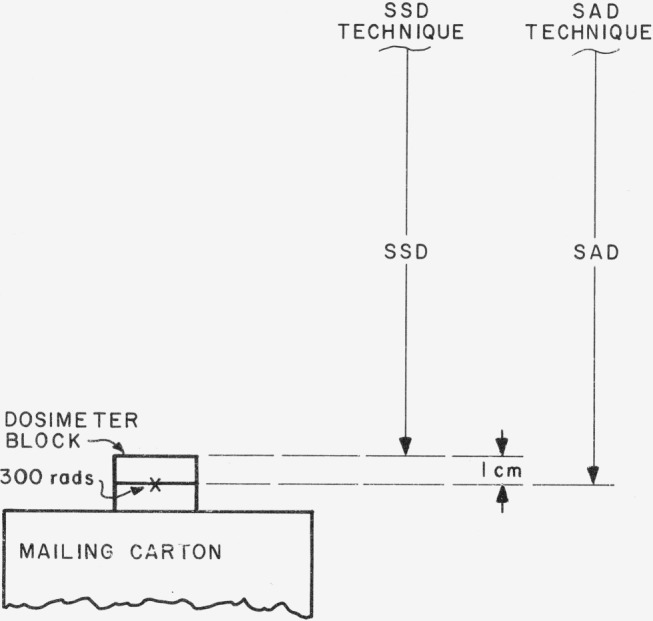
Prescribed irradiation geometry. Participant is to employ his usual distance and technique (either source-to-surface, SSD, or source-to-axis, SAD).

Six dosimeter blocks per beam, five for irradiation and one as a control, are being mailed to the radiotherapy departments that expressed their willingness to participate in the study. Along with the dosimeters go instructions for their irradiation, and exposure-information forms to be completed and returned with the dosimeters. Figure 1 gives the essentials of the prescribed irradiation geometry. Using the mailing carton as support, the participants are asked to irradiate the dosimeter blocks identically, one at a time, using a 10 cm × 10 cm field and their usual distance and technique. Although no large phantom is employed in order to avoid bulk in mailing, they are asked to compute irradiation time to deliver 300 rads (3.0 Gy) to water at a depth of 1 cm in a large water phantom. The same procedure is followed by NBS in the calibration of the dosimeters. The choice of 300 rads to be delivered to a large water phantom was made to approximate realistic therapy conditions. The depth of 1 cm was chosen simply for convenience. It is expected that the dose at this depth (which is just beyond the depth of maximum dose delivered) may be derived to within 1 percent from that of the customary 5-cm depth by means of available depth-dose data [1].^1^

The main exposure-information form, which readily can be completed by a technician, requests time and date of irradiation, distance, field size, irradiation time, and identification of the operator and the person supplying the information. An optional form, to be filled out by the physicist, asks for information regarding source calibration, for use by NBS in an effort to assist the participants in pinpointing the cause for any significant deviations of the NBS dose interpretation from 300 rads.

Upon completion of dosimeter evaluation in terms of absorbed dose to water, the participants included in a given mailing are informed of their own results only and of their standing among their peers. For participants completing the optional information form regarding source calibration, NBS further furnishes a value for the absorbed dose computed from the calibration information. Performance of all participants is known only to NBS and is treated as confidential information.

## 3. Description of Dosimetry System and Method of Dose Evaluation

### Choice of Dosimetry System

TL systems have proven their value in several past survey studies involving the mailing of dosimeters [2]. For the present repetitive operation, the main considerations guiding the selection of a TL system are ruggedness, ease of handling and maintenance, and a relatively small dependence of TL response on irradiation and thermal histories. Considerations of fading characteristics are of minor importance since fading corrections can be applied readily. So are considerations of dependence of response on photon energy. For most source geometries encountered in medical ^60^Co gamma irradiators the fraction of scattered photons with energies below 100 keV, to which the high-atomic number CaF_2_:Mn detector would show a relatively high response, is expected to be negligibly small compared with the total scatter [3]. At the core of the dosimetry system are commercial quartz bulbs, each containing two pieces of pressed crystalline CaF_2_:Mn in contact with a metallic heater strip, and a commercial assembly consisting of a bulb-heating unit and an electronically cooled photomultiplier. The rest of the system consists of parts assembled in the laboratory specifically for use in the present application.

**Figure 2:**
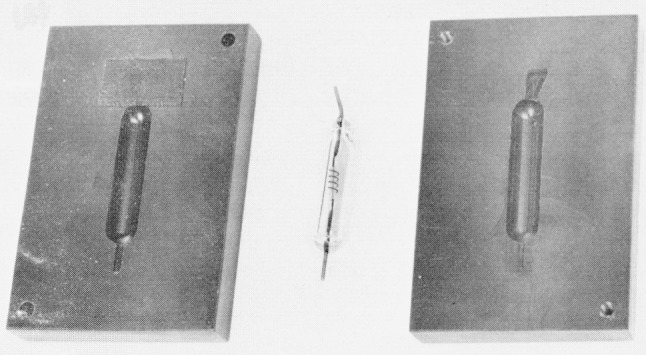
The chosen thermoluminescence dosimeter. The dosimeter bulb is placed into one of the halves of a black polystyrene block, each half having the dimensions 7.6 cm × 5.0 cm × 10 cm. The halves are screwed together with Nylon screws. A tight fit of the bulbs is assured by tape over the cutout for the bent pin in one of the block halves. During irradiation, the block surface bearing the recessed screw heads faces the radiation source.

During shipment and irradiation, the TL bulbs are cradled in black polystyrene blocks (see fig. 2). The blocks provide shielding from prolonged influence of visible light, to which CaF_2_:Mn is not entirely insensitive, and maximum electron buildup in polystyrene at the bulb surface in a ^60^Co gamma-ray field. One of the bulb pins is seen to be bent. This fixes the orientation of the bulb during irradiation and readout, which was found to be mandatory for good reproducibility, particularly in radiation fields containing an appreciable low-energy component. For the present study, the pins were bent in such a way that, during irradiation, neither of the two CaF_2_:Mn pieces is shielded from the direct photon beam by the central heater strip. For flexibility in the selection of readout parameters and data handling not provided in the commercial bulb reader, a current integrator is used that permits either direct printout of the data or interfacing with a teletypewriter and paper-tape printer. The assembly is controlled by an electronic timer in a circuit designed to permit independent variation of total heating and current-integration times.

### Dosimeter Calibration

Prior to each mailing, each dosimeter is individually calibrated in a two-stage procedure. In the first stage, the relative response of the individual dosimeters is determined at low exposure levels (3–5 R), with the time intervals between irradiation and readout adjusted to eliminate the need for fading corrections. The ten relative calibration sequences administered prior to the start of the program demonstrated relative standard deviations for the reproducibility of repeated individual dosimeter readings ranging from about 0.1 to 2 percent, with an average of 0.8 percent. Additional relative calibration sequences administered to each dosimeter between any two mailings were found to be necessary since response of some dosimeters decreases with use, while others it remains relatively constant.

The second stage consists in an absolute calibration with ^60^Co gamma rays, covering an absorbed-dose range from about 100 to 600 rads in water in a geometry identical to that used by the participants. Absorbed dose at a 1-cm depth in water is computed from exposure using accepted methods and parameters [4, 5, 6]. The calibration dosimeters are read out along with the dosimeters returned by the participants, and with shipping and laboratory controls. The slightly supra-linear function of dosimeter response-versus-exposure, obtained by least-squares fit, forms the basis for the dose interpretation from the dosimeter response of the unknown samples.

### Correction for Residuals

In experiments carried out in the course of the preparations for the present survey, we found indications that, at a given time after initial irradiation and readout, there exists a residual level that is characteristic for the particular exposure. When the exposure level is changed, the residual level characteristic for the new exposure establishes itself gradually upon repeated irradiation and readout. Because of these findings and in view of the results of Lucas and Kaspar [7] regarding a possible delayed transfer to shallower (accessible) traps of electrons initially in traps that are inaccessible to a particular TL readout, determinations of residuals were carried out for the level and the time sequence actually employed in the present study. The results confirmed the need for small corrections to the 3–R readouts employed in the determination of relative dosimeter response after readouts of high exposures, the residual response after the readout of a 400–R exposure being between 0.5 and 1.5 percent of the response to a 3–R exposure. No correction for residuals is required for readouts of exposures above ~100 R.

### Correction for Increase in Photomultiplier Sensitivity During Extended Readout Periods

The two types of photomultipliers tested (one a relatively insensitive copper-beryllium dynode tube, the other a sensitive bialkali-dynode tube) both exhibit gain changes with repeated use. For the present study, the sensitive bialkali-dynode tube is employed, at a voltage leading to current signals of about 10/*μ*A for the TL response to 300 R. Under these operating conditions, the photomultiplier gain increases monotonically by almost 4 percent, probably due to insufficient cooling at high signal levels, during the roughly 3 h required for the readout of about 270 dosimeters, of which about 200 received ~300 R. A suitable correction for this increase is obtained with the aid of readings taken at regular intervals during the readout sequence of the constant light source built into the reader.

### Choice of Readout Technique

Since the TL dosimetry system employed relies on annealing of the CaF_2_:Mn material solely during the readout cycle, a readout technique was chosen that enables one to mix irradiations and readouts over the entire contemplated range without appreciable interference from incandescence or from release of residual trapped electrons. Yet, to prolong the life of the dosimeters, heating power is kept at a minimum. An annealing time of 21 s is used throughout. For readout of dosimeters mailed to the participants, and of all associated calibration dosimeters receiving exposures of 100 R or more, the heater current is 6 A, producing close to maximum thermal load. For readout of the low-level (3–5 R) exposures given in the course of the determination of relative dosimeter response, a heater current of 5.5A is sufficient. Signal-integration times of 14 s and 16 s for the 6–A and 5.5–A readouts, respectively, are used since they have been found to produce maximum discrimination against incandescence, while including most of the glow peaks.

### Corrections for Fading

The reduction in the TL signal was found to be about 4 percent over the decade between 20 and 200 h, while it was only about 2.5 percent over the subsequent 500 h. Consequently, the correction for fading applied to the readings of the dosimeters exposed by the participants varies between about 4 and 6 percent, for the usually encountered 170-to 700-h delay between irradiation and readout.

## 4. Analysis of Uncertainties in the Dose Interpretation from TL Response

A valid assessment by NBS of the participants’ ability to deliver a prescribed absorbed dose necessitates a knowledge of the uncertainty inherent in the NBS procedure for determining absorbed dose from dosimeter response. In table 1, averages derived from two or more of the past four mailings are given for estimated upper bounds for the uncertainties due to the systematic and random errors inherent in the NBS procedure for determining absorbed dose. The uncertainties due to the systematic errors in the location of the response and fading curves were obtained from the statistical information for the least-squares fits of these curves as three times the standard deviations of the predicted values in the ranges of interest. The uncertainty due to all systematic errors then was taken to be the sum of the individual uncertainties listed. The hard-to-assess uncertainties associated with the generally accepted values of the parameters and constants used in the computation of absorbed dose from exposure were not included.

In the evaluation of the uncertainties due to the random errors, the argument was used that the sources for the random errors in the absorbed-dose determination from the response of the dosimeters irradiated by the participants are essentially the same as for the evaluation of the calibration and fading data. Using this argument, the uncertainty due to the random errors shown in table 1 for the dose evaluation from the response of a single dosimeter was computed by combining 3 times the standard deviations associated with the least-squares fits of the calibration and fading curves in quadrature, after suitably weighting by the degrees of freedom for each curve fit. The corresponding uncertainty of the results from readings on five dosimeters then was obtained by division by $\sqrt{5}$ finally leading to an estimate of about 4 percent (or ± 12 rads, at the 300-rad level) for the algebraic sum of the uncertainties due to the total systematic and random errors. This is the total uncertainty of the NBS dose interpretation from the average response of five dosimeters.

## 5. Discussion of Results of the First Four Test Series

### NBS Dose Interpretation from Response of Dosimeters Irradiated by the Participants

**Figure 3:**
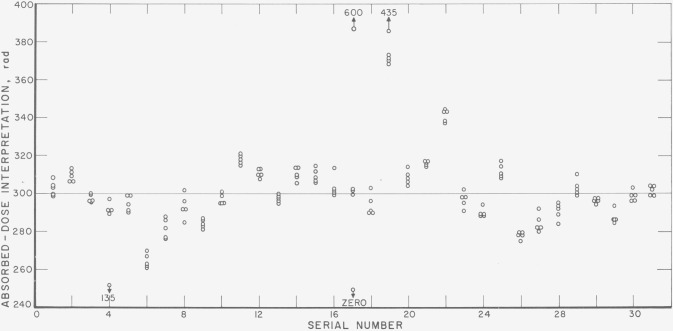
NBS dose interpretations from individual dosimeter response for a typical mailing batch.

Figure 3 gives the dose interpretation for the individual dosimeters submitted for irradiation by the participants in one of the past mailing batches. In view of the analysis given above for the uncertainties in the dosimetry method, differences of ±5 percent in the average dose interpretation must be considered significant. Of the 33 sets for which results are shown, ten are outside the ±5-percent limit (one of these, number 17, probably because one of the dosimeters was irradiated twice by mistake, while another presumably irradiated dosimeter remained blank). It is of interest to note that, in most instances, the readings on the individual dosimeters are within the ±3 percent uncertainty due to the random error of the NBS dose interpretation from individual dosimeter readings, the most pronounced exceptions being observed in sets number 4 and 19.

**Figure 4:**
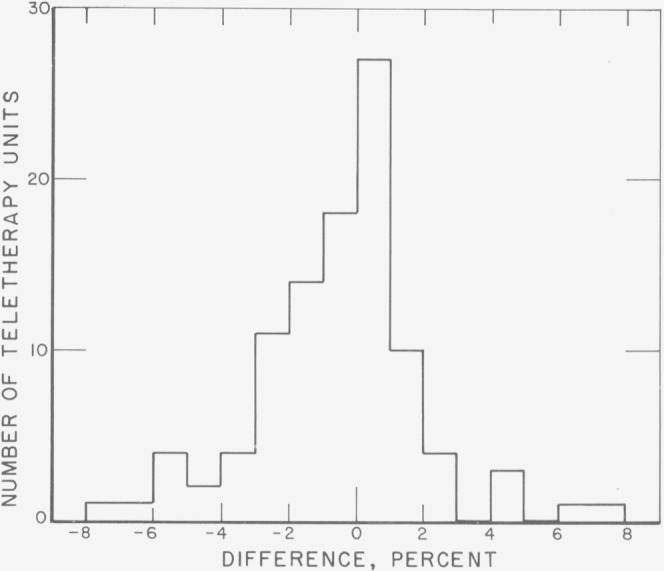
Survey, results of first four mailings. Shown is the percent difference between the dose presumably delivered and the NBS dose interpretation from the average response of the five dosimeters in each set. The percentage intervals are closed at the upper bounds. In the cases in which one dosimeter gave a significantly different dose interpretation from the other four, the interpretation from this dosimeter is omitted from the average. The results from the one set for which two of the five dosimeters gave significantly different dose interpretations are omitted altogether. The two discrepancies of around 60 percent probably are due to a misunderstanding by the participants regarding the absorbed dose to be delivered.

The results for the first four mailing batches are shown in figure 4 in the form of a histogram giving the difference, in percent, between 300 rads and the average NBS dose interpretation. Of a total of 114 sets, 85 (or about 75%) show results differing from 300 rads by less than 5 percent; another 22 (or about 20%) show differences of between 5 and 10 percent; there are 4 with differences over 20 percent.

### Sources of Error in Delivered Dose

Only through laboratory visits is it possible to isolate all sources of error in actual dose delivery. The NBS study does not involve any laboratory visits. In fact, only during the initial pilot mailing comprising the facilities of the U.S. Veterans Administration and the U.S. Public Health Service was it possible to obtain information on some of the sources of error either by direct or indirect personal interchange. What was learned through this interchange was, e.g., that timer malfunction had been observed previously by the participants in two out of the three cases in which a spread in individual readings large compared to NBS uncertainty due to random error had been observed on presumably identically irradiated dosimeters. Also, a discrepancy of more than 20 percent was traced by one participant to an error in the computer output upon which his current dose-rate chart had been based.

**Figure 5:**
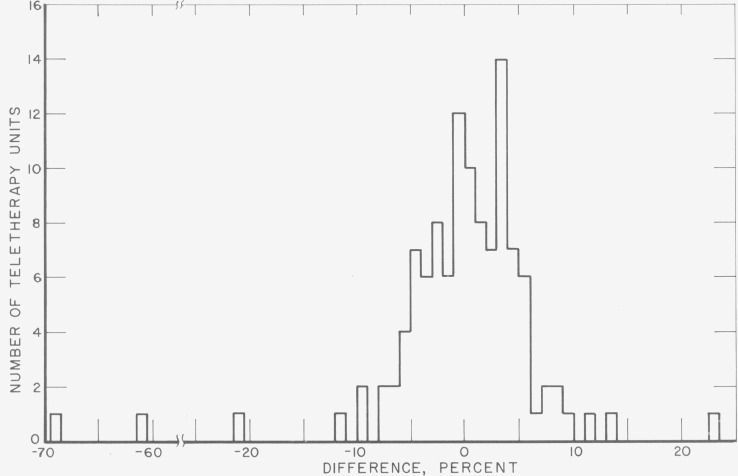
Check on participants’ computations. Shown is the difference between the participants’ computation and the NBS computation check, made with the aid of the information supplied by the participants. The percentage intervals are closed at the upper bounds. Differences of 1 percent or less are not considered significant.

In all but the initial mailing, performance analyses were derived from information provided in the supplied forms, a method that makes it impossible to isolate errors due to faulty source calibration from errors of timing or positioning. However, discrepancies due to errors in dose computation are readily apparent from an analysis of a participant’s exposure factors as compared to the source-calibration factors supplied. Figure 5 is a histogram showing a break-down of the difference between 300 rads, and the NBS computation for the sets for which the information received was sufficiently clear. In somewhat more than one-half of the 96 cases covered, the difference is seen to be larger than 1 percent, 8 cases showing differences between 5 and 8 percent. For the 53 sets covered in the first two mailings, a detailed analysis was made of the types of error in the participants’ computations. Table 2 shows the results, indicating that most errors arise from a failure to apply one or the other factor in the computation of absorbed-dose rate from the calibration data, but that there are a number of other errors, as well. While no detailed analysis of the types of computational errors was performed for the participants in the later mailings, a cursory inspection revealed in one instance a division instead of multiplication by the depth-dose correction factor, and in another instance the application of the same depth-dose correction factor twice, in successive stages of the computation.

### Frequency of Source Calibration

Another interesting item gleaned from the information supplied by the participants is shown in table 3, giving a break-down for the 72 sets for which information was available on the time elapsed between the last source calibration and the dosimeter irradiation. In spite of the NBS instructions not to use any special handling or irradiation procedures for the dosimeters, a relatively large number of participants evidently did a special calibration immediately prior to dosimeter irradiation. In fact, in some instances the dosimeters were irradiated by or at least under the supervision of a medical physicist, a procedure that was in contradiction to the instructions mailed with the dosimeters. So far, no correlation is evident between performance and time elapsed since source calibration.

## Figures and Tables

**Table 1:** Uncertainty in determination of absorbed dose from TL response

Sources of error	Resulting uncertainties	
*Systematic*		
NBS exposure calibration of ^60^Co gamma-ray source ^8^	0. 7%	
Location of TL response-versus-absorbed dose curve	. 7	
Location of fading curve	1. 0	
Positioning of dosimeters during calibration	. 1	

Total		2. 5%
*Random*		
Evaluation from single dosimeter response from average response of five dosimeters	3. 0%	
		1. 3
*Total uncertainty in dose interpretation from average response of five dosimeters*		~4%

**Table 2:** Type of computation errors greater than 1 percent encountered in the first two mailing batches. (Total number of sets covered: 53)

Type of error	Frequency

Omission of factors in derivation of absorbed-dose rate:	
Inverse-square correction	13
*f* factor (quotient of absorbed dose in water under equilibrium conditions over exposure at the same point)	7
Attenuation factor (also referred to, e.g., as “cap correction factor”)	14
Back-scatter factor	12
Percent depth dose	1
Decay correction	2
Use of inverse-square correction where none was required	1
Use of numerical values other than those considered up-to-date by NBS^a^	3
Errors in arithmetic	3

a Not including differences due to the use of *f* for water rather than tissue.

**Table 3:** Time elapsed since participants’ last source calibration. (Total number of sets covered: 72)

Time elapsed	Number of sources

Less than 1 week^a^	17
Between 1 week and 3 months	27
Between 3 and 6 months	11
Between 6 months and 1 year	11
About 1½ years	2
About 3 years	1
About 4 years	1
About 5 years	2

a 13 of them less than 1 day.

## Footnotes

1 Figures in brackets indicate literature references at the end of this paper.
